# Protocol for A randomised feasibility trial comparing fluoride interventions to prevent dental decay in older people in care homes (FInCH trial)

**DOI:** 10.1186/s12903-021-01650-9

**Published:** 2021-06-14

**Authors:** Rakhee Patel, Iftekhar Khan, Mark Pennington, Nigel B. Pitts, Claire Robertson, Jennifer E. Gallagher

**Affiliations:** 1 Faculty of Dentistry, Oral and Craniofacial Sciences, Dental Public Health, Centre for Host Microbiome Interactions, King’s College London, Denmark Hill Campus, Bessemer Road, London, SE59RS UK; 2 Clinical Trials Unit, University of Warwick, Gibbets Hill, Coventry, CV1 7AL UK; 3 Medicines and Health Regulations Agency (MHRA), Canary Wharf, London, E1 UK; 4 Centre for the Economics of Mental and Physical Health, Institute of Psychiatry, Kings College London, De Crespigny Park, London, SE58AF UK; 5 Faculty of Dentistry, Oral and Craniofacial Sciences, Dental Innovation and Translation Centre, King’s College London, Guys Hospital Campus, London, SE1 9RT UK

**Keywords:** Feasibility trial, Care homes, Older people, Fluoride intervention, Nursing, Residential

## Abstract

**Background:**

The number and proportion of older people globally is growing faster than that of any other age group. At the same time the number of people retaining some of their own teeth is rising. There significant differences between those living in care and their community dwelling peers, with evidence showing those in care having fewer teeth and significantly higher levels of dental decay. There are numerous Cochrane reviews linking the use of fluoride to a reduction in dental decay, however, the majority of research on effectiveness has been conducted on children and consequently, children and adolescents tend to be the main recipients of fluoride interventions. There are to date no studies comparing the effectiveness of fluoride interventions in older people in care homes in the UK. However, prior to developing an appropriate protocol for full-scale trial comparing clinical effectiveness of fluoride interventions, there are a number of trial feasibility and statistical parameters that need to be clarified.

**Methods:**

This trial is a single centre, multi-site randomised controlled assessor blind parallel group (three groups) trial, with the primary objective of establishing the feasibility, practicability and compliance of fluoride interventions to prevent dental decay in care homes. Secondary and tertiary objectives will aim to explore the acceptability of the interventions from resident, care home and dental services perspectives, and estimate the efficacy of the three different fluoride treatments.

**Discussion:**

This feasibility trial will produce new knowledge and add value to a landscape that is under researched. Although the efficacy of fluoride interventions is proven, the feasibility of dental research and prevention in this vulnerable group and in the complex care home setting is novel. This work will not only add to our understanding of the interface of dental care and social care but will also contribute to our broader understanding on undertaking research in care home settings. Dental care for older people has been a longstanding issue, and the events of this past year has shone a light on the vulnerabilities of those residing in care homes and so this research is landing at a pivotal time.

*Trial registration* EudraCT Registration 2017-002248-34. Registered 20th February 2018 https://www.clinicaltrialsregister.eu/ctr-search/search?query=2017-002248-34.

## Background

Increased life expectancy and reduced mortality, coupled with a falling birth rate mean that the proportion of older people is growing faster than that of any other age group globally. As a greater proportion of the population survives to very old ages, the public health impact of the burden of disease, disability and related utilisation of medical care and the need for supportive and long-term care has become an important concern. As the population profile changes, it is predicted that the number of people in care will also rise.

As well as the number and proportion of older people increasing, at the same time the number of people retaining some of their own teeth is rising. In the UK 2009 Adult Dental Health Survey, 53% of people surveyed over the age of 85 years had natural teeth, with an average of 14 teeth [1]. This is leading to a shift in oral health from an older population, which was largely edentulous (i.e. had no natural teeth) to one, which is dentate and so can benefit from prevention, restoration and maintenance of the dentition.

Poor oral health impacts an individual’s ability to eat, type of diet, weight, speech, appearance and social interaction. Restriction of diet related to issues with chewing or swallowing, dry mouth and pain can cause malnourishment and nutritional deficiencies in older people [2]. Evidence shows that nutritional deficiencies in older people is linked with excess winter deaths, cardiovascular disease and stroke [2], and so oral health in older people is important. Evidence has also shown an association between the presence of oral bacteria and pneumonia in older people in care homes [3]. Older people perceive oral health as being important to their quality of life in a number of different ways, including function, general health socialising and confidence [4].

The combination of frequent sugar intake, poor oral care and medications that affect salivary flow, means that older people in care homes are at a significantly higher risk of dental decay [5]. Epidemiological studies have shown that the incidence of root and coronal decay increases with age [6] and that older adults are at higher risk of decay than children [7].

Steele and Walls [8] showed significant differences between institutionalized and free living older people, with those in care having fewer teeth and significantly higher levels of dental decay. A study undertaken in Avon, found that 63% of residents in nursing care had root decay at the time of examination [9]. Similarly, high levels of unmet needs have been found internationally with residents requiring extensive dental treatment due to poor oral health [10, 11].

The poor oral condition is exacerbated by a lack of access to dental services and treatment options. Most homes access dental care only when the resident has a problem [12]. In addition, when a need for dental treatment is identified, in many cases the complex challenges that present in these patients such as medical complications, limited mobility and compliance, means the range of dental treatments deliverable is restricted.

These factors dictate a need for prevention of dental disease in this population. Dental prevention should be targeted at susceptible tooth surfaces in the most vulnerable members of the population. There is a strong body of evidence linking the use of fluoride to a reduction in dental decay [13, 14]. The majority of research on effectiveness has been conducted on children and consequently, children and adolescents tend to be the main recipients of fluoride interventions. However, more recently studies have shown fluoride to be effective in reducing dental decay in older people [15].

There are many modes of fluoride delivery available, however considering the complex challenges that present in older people in care, the two most appropriate technologies in the care home setting would be professionally administered fluoride varnish and high dose fluoride toothpaste. These would be the safest modes of fluoride delivery as a controlled dose can be administered.

A review of the evidence pertaining to the use of fluoride supplements in older people in care homes concluded there is good evidence of dental decay reduction with 22,000 ppm fluoride varnish professionally applied 3–4 times per year [16]. A randomized control trial undertaken in care homes in Hong Kong showed 3 monthly applications of fluoride varnish by a dental professional to be more effective in reducing root caries than with oral care training of care staff alone [17].

Fluoride delivery through regular brushing is a simple and effective decay preventive intervention, and is part of the ‘activities of daily living’ within care home standards. There is a dose related response to decay rates with fluoride, and commonly people deemed ‘high risk’ of dental decay are advised to use a high dose fluoridated toothpaste [18]. Over the counter toothpastes contain a fluoride does of between 1000 and 1450 ppm. There are 2 commercially available high dose toothpastes available in the UK, with fluoride doses of 2800 ppm and 5000 ppm. A resident level randomized control trial undertaken in care homes with older people showed a significant reduction in root decay in participants using 5000 ppm fluoride toothpaste compared to 1450 ppm fluoride toothpaste over an 8-month period [19, 20]. This study was undertaken in Denmark, where the 2800 ppm fluoride toothpaste is not available.

In their literature review of fluoride interventions in older people in residential care homes, Innes and Evans [16] concluded that although 5000 ppm fluoride toothpaste is effective in reducing dental decay in elderly people in care homes, older people may swallow more toothpaste than a younger person due to lack of supervision, poor oral function or confusion it would seem prudent to recommend 2800 ppm toothpaste to avoid any risk of fluoride overdose. However, no studies comparing the effectiveness of 2800 ppm to usual care (1450 ppm toothpaste) in a care home setting have been carried out to determine the extent of impact on dental decay, although studies in children and adults have demonstrated effectiveness.

There are to date, no studies comparing the effectiveness of fluoride interventions in older people in care homes in the UK. However, prior to developing an appropriate protocol for full-scale trial comparing clinical effectiveness of fluoride interventions, there are a number of trial feasibility and statistical parameters that need to be clarified.

Dental decay in older people in care homes poses a significant impact on the National Health Service and society and yet is entirely preventable. Development and implementation of a fit for purpose fluoride intervention as part of routine care for older people in care could impact significant public health benefits by improving the oral health of residents in care homes and consequently their general health and quality of life. However, decision making on which intervention would be most suitable is hindered by the limited research undertaken in care homes on the effectiveness of 2800 ppm fluoride toothpaste, its comparison to professionally administered fluoride varnish. Consequently, this research is of great importance to health improvement and policy setting.

## Methods

### Trial objectives

The aim of this randomised feasibility study is to establish and compare the feasibility of fluoride programmes to prevent dental decay in older people in care homes.

- *Primary objective* To establish the feasibility, practicability and compliance of fluoride interventions to prevent dental decay in care homes.
- *Secondary objectives* To explore the acceptability of the interventions from resident, care home and dental services perspectives, and the impact on of the interventions on resident’s quality of life.
- *Tertiary objectives* To estimate the effects of 3 different fluoride treatments in the prevention of dental decay in older people residing in care homes. To provide preliminary data on the economic value of the three interventions to the patient or NHS payer.

To assess the feasibility, a number of in priori *criteria* have been set as broad indicators. These are:

*Criterion 1*: Recruitment, retention and attrition

1. > 50% positively screened for inclusion
2. Resident recruitment rate to trial of at least 60%
3. Attrition rate of < 35%

*Criterion 2*: Compliance, acceptability and fidelity

1. Randomisation, intervention delivery and data collection methods are acceptable to the majority of residents, family members and care staff
2. Care staff will deliver oral care where possible to residents, and record this accurately in the personal care plan and drug charts.

*Criterion 3*: Statistical considerations and costs

Measurement variability and standard deviation is such that the sample size determined for the full-scale trial is achievable within reasonable costs and resources.

### Trial design and setting

This feasibility study will be a single centre, multi-site cluster randomised controlled assessor blind parallel group (3 groups) trial, with a nested qualitative component.

**Fig. 1:**
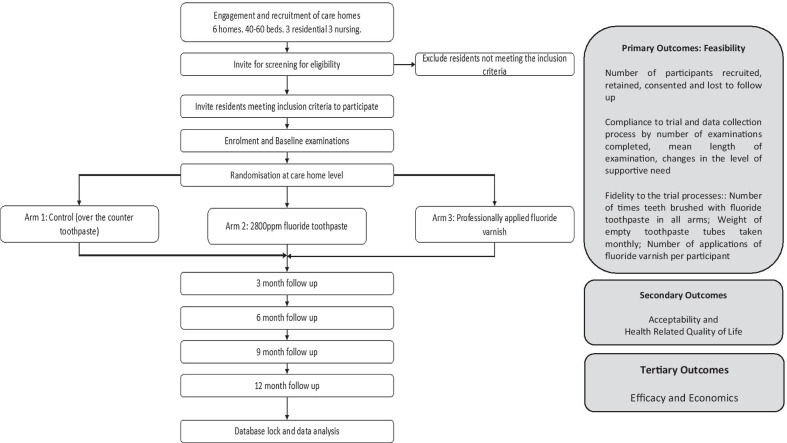
FInCH trial flow diagram

A flow diagram for the trial is provided in Fig. 1. Intervention delivery and follow up will be over 12 months and will be conducted in six London care homes housing between 60 and 80 residents. The sample will be stratified to attain balance in each arm between nursing and residential care.

Within this, the nested qualitative component of the trial will gain views from care home managers, carers, residents and family members as well as the dental team. These views are vital in assessing the acceptability objective of the study.

### Ethical considerations

The ethical considerations around this trial have been carefully thought through. Undertaking a clinical trial with a vulnerable population, who may not be able to consent in many cases requires careful planning and a considered approach to ensure residents are protected at all times. Informed written consent to participate in the trial from the resident or legal power of attorney will be sought for all eligible residents following extensive consultation with the care home, residents and family.

In considering risk relating to the investigational medicinal product (Duraphat® 2800 ppm and varnish) risks to participants would be no higher than that of standard medical care (classified a type A based on MRC/DH/MHRA Joint Project 2011). The licensed medicinal products being used will be used for their licensed range of indications (prevention of dental decay), dosage and form in this study, and so this has been determined as a Type A low-risk trial.

Favourable ethical approval for the FInCH trial was granted in February 2018 by the East of England—Cambridge South Social Care Research Ethics Committee (reference 17/EE/0475).

Monitoring of this trial will be to ensure compliance with Good Clinical Practice and scientific integrity will be managed and oversight retained, by the King’s College Hospital Clinical Trials Quality Team. As per the regulations, annual progress reports, notification of End of Trial, and a final report at the conclusion of the trial will be submitted to the Research Ethics Committee (REC) and to the Medicines and Healthcare products Regulatory Agency (MHRA).

### Care home recruitment

Participants will be recruited from six care homes offering residential and nursing care in one outer London borough.

A programme of engagement will be undertaken with each home and with the support of the local authority care homes team.

Carers and management staff will be invited to interviews and focus groups and the approved information leaflet and consent forms will be presented at staff meetings.

The study will be introduced to all care home residents via posters and presentations at family care home meetings. If the resident is unable to consent, their legal representative will be approached for recruitment, be that a family or friend with the residents power of attorney, or the care home. All relevant and approved documentation including the patient information leaflet, family information letters and written consent forms will be provided to the participant, and their legal representative so an informed decision to participate can be made. Opportunities for discussion will be provided.

### Study participants

1. Care home residents

All residents, male or female, over the age of 65 years will firstly be invited to participate to a screening to assess if they meet the criteria by the chief investigator. Once informed consent is in place, be it from the resident or their legal representative, the resident will be screened by the researcher (RP) to assess eligibility for the study trial based on the inclusion and exclusion criteria.

If the resident is eligible to participate, consent forms for trial participation will be signed and within 28 days of the residents screening appointment, and the participants baseline examination will be undertaken.

It will be made clear that participants have the right to withdraw from the study at any time for any reason. In addition, the investigator also has the right to withdraw patients from the study treatment in the event of inter-current illness, adverse events or other reasons of concern.

Participants that withdraw will not be replaced. Levels of attrition are part of the objectives of this trial, and if a participant withdraws, all efforts will be made to report the reason for withdrawal as thoroughly as possible.

2. Care home staff

Carers and management staff from the homes will be invited to participate in interviews and focus groups to explore their perspective on the study objectives and processes. Sessions will be guided by a topic guide informed by the literature and trial objectives. The topic guide will cover domains on perceptions of oral health, delivery of mouth care, challenges, facilitators, access to dental care and training. Details of the Information leaflets and consent forms will be provided in advance of the meetings via staff meetings. Clarification will be provided to staff in advance of obtaining consent. All interviews and focus groups will be audiotaped and transcribed by a confidential transcription service for data analysis. Participants will be able to withdraw at any time without giving a reason but will be informed that they are unable to withdraw data from focus group discussions to retain the integrity of the discussions.

### Eligibility criteria

#### Inclusion criteria

- Residents (male or female) over 65 years residing in one of the enlisted care homes
- Residents with at least 5 natural teeth or roots
- Provision of written informed consent by the resident or residents legal representative

#### Exclusion criteria

- Residents who are not able to consent themselves, and have no registered legal representative and therefore valid consent cannot be sought
- Hypersensitivity to the active substance, colophony or to any of the excipients of the IMP or comparators
- History of active ulcerative gingivitis, active stomatitis and bronchial asthma.
- Residents with facial or oral infections e.g. Cold sores or draining sinus.
- Residents at the care home on end of life care
- Females of childbearing potential. Fluoride varnish is contraindicated to pregnant women therefore all female participants will need to be at least one year post-menopausal.

### Trial interventions

#### Arm 1: Control

The control group will be subject to usual care practices with staff training. This will include using an over the counter fluoride toothpaste. To ensure consistency of dosage in the control group, the control group will be provided with Colgate Total 1450ppm (0.32%), which they will be advised to brush their teeth with a 1cm line of the toothpaste twice daily which equates to a fluoride dose of 1.16mg per application.

#### Arm 2: 2800 ppm fluoride toothpaste

Duraphat® 2800ppm (0.619%) fluoride toothpaste has been chosen as it is commonly prescribed high dose toothpaste in the UK and has been used in previous clinical trials. As per the manufacturers recommendations, a 1cm line is to be used twice daily instead of the normal toothpaste, which equates to 2.24 g fluoride per application. Each tube will be labelled with directions of use and the patients details. Staff will be trained with these recommendations and advised to cease use if any adverse effect occurs, and report this to the research lead immediately.

#### Arm 3: Fluoride varnish

The varnish used will be Duraphat® 50 mg/ml dental suspension, produced by Colgate-Palmolive Ltd. This varnish has been chosen as it is the most commonly available and used fluoride varnish in the UK and has been used in previous clinical trials.

The fluoride dose in the varnish is equivalent to 22,600 ppm. As per manufacturer recommendation, before applying Duraphat®, excess debris will be removed from the tooth surface and the teeth dried. The varnish will be applied as a thin layer to the most susceptible areas of dentition using a brush. The dose administered will not exceed 0.75/ml (= 16.95/mg Fluoride). As the residents are deemed high risk, this will be repeated every 3 months for the duration of the study (within 2 weeks of baseline examinations, 3 months, 6 months, 9 months).

A suitable trained and qualified dental hygienist will administer the varnish. Treatment record forms will be used to document date, batch number, patient identification number and number of teeth varnish was placed on.

To ensure consistency with the control group, patients in arm 3 were provided with Colgate Total 1450 ppm (0.32%), which they will be advised to brush their teeth with a 1 cm line of the toothpaste twice daily which equates to a fluoride dose of 1.16 g per application.

### Prescribing of medicinal products

Duraphat® toothpaste and varnish are licensed, prescription only products in the United Kingdom. Both products are licensed for use for the prevention of dental decay, which is the purpose it will be used for in this study. Experienced, qualified clinicians will be administering/recommending the interventions in this study, which are relatively low risk.

As this is a community intervention, the Community Dental Service Clinical Director/Dental Public Health Consultant, will put a patient group directive in place for all identified residents, in accordance with current regulation and practice. This is the route followed for dental community based interventions, and so will be reproduced in this study.

Under this overall care home prescription, a visiting dental care professional place fluoride varnish to those consented, and record this in the treatment record form. As the fluoride varnish in this trial is a marketed products to be administered in accordance to the indication specified by the marketing authorisation interpretation of Regulation 46 of Statutory Instrument 2004 No. 10 1 suggests that the labelling requirements of Article 15 of Commission Directive 003/94/EC(ar) does not apply in this case, and so varnish tubes will be not need to be individually labelled.

For the toothpastes, dispensing will be as per standard of care and therefore the study drugs will not require labelling in line with annex 13. The IMP and comparators will be managed as per standard of care and will not require special labelling, accountability or storage. We anticipate each resident will need a maximum of a tube per month of whichever toothpaste they are allocated, as this is the suggested amount for a fully dentate individual. Following the clinical examination and checking of the medical history, the dentist will give a one-month supply of toothpaste labelled with the resident’s name and date of birth to the corresponding resident or carer. Each month the researcher will visit the home and weigh the tubes to assess compliance to use of the IMP, and give additional supplies as needed. As randomisation is at the care home level, it is not felt that blinding of toothpaste tubes is needed, nor is it possible for the varnish arm of the trial.

### Outcome measures

#### Primary outcome: feasibility

- Number of participants recruited, retained, consented and lost to follow up (drop out) through the trial period in each trial arm.
- Compliance to trial and data collection process by number of examinations completed, mean length of examination, changes in the level of supportive need (as measured by the indicator of relative need ‘IoRN’ tool [21]) through the trial period in each trial arm.
- Fidelity to the trial processes:
  1. Number of times teeth brushed with fluoride toothpaste in all arms
  2. Weight of empty toothpaste tubes taken monthly
  3. Number of applications of fluoride varnish per participant

#### Secondary outcomes: acceptability and Health Related Quality of Life (HRQoL)

- Acceptability endpoints
  1. Acceptability and preference for the intervention arms via in depth interviews with residents through the trial period in each trial arm.
  2. Organisational acceptability via focus groups with carers and in depth interviews with care home managers and community dental services.
- HRQoL endpoints

Impact on HRQoL for residents will be assessed using reported frequencies of the oral impacts on daily performance survey tool and the EQ-5DL tools

#### Tertiary outcomes: efficacy and economics

1. Efficacy outcomes: Number and proportion of teeth with decay progression at tooth surface level between trial arms
2. Number of teeth with decay arrest at tooth surface level
3. Number of teeth with no change in decay at surface level
4. Economic Outcomes:
  The following outcomes will provide preliminary evidence for the cost-effectiveness of the intervention
  1. Use of health resources relating to dental care (general dental or medical practitioner visits, domiciliary care visits, out of hours’ emergency dental center visits) for the intervention year versus the year preceding the study collected from resident’s care records
  2. Use of health resources for delivery of the intervention (dental team hours, resources, medicinal products) from data collection forms completed by dental team.
  3. Use of resources in the home (carers time, administrative hours) from data collection forms completed by care team
  4. Compare the above between the trial arms

### Randomisation

Randomisation will be at the care home level. (i.e. site level).

Residents will be screened for eligibility at each site, and demographic data (age, gender, level of supportive care) will be collected. This will then be used to assess distribution of participant characteristics between the arms, and if a minimisation algorithm needs to be used. This will minimise for age (65–84 vs 85 +), gender (male vs female) and level of supportive care (nursing vs residential) where possible.

Randomisation service will be provided by The UKCRN registered Institute of Kings Clinical Trial Unit. Randomisation will be to a 1:1:1 ratio, undertaken within 7 days of the final baseline resident examination.

As the trial is an open label no emergency code break is required.

### Follow up of participants

Participants will be followed up over 12 months (Table 1).

**Table 1:** Trial procedures by visit

Visit	Procedures to be performed
Screening	Complete process for written informed consent with resident or legal representative (including mental capacity assessment)
	Gather demographic information
	Complete medical history
	Review concomitant medications
	Brief oral examination
	Eligibility assessment
Baseline examination (within 28 days of screening)	Changes to mental capacity
	Examination using ICDAS root caries scoring system undertaken by blinded assessor
	Review concomitant medications
	Adverse event monitoring
	Undertake in depth interviews with residents to consider acceptability, QoL (OIDP and EQ5D5L and EQ5D3L survey tool) and relative need (IoRN2)
	Compliance check
	IMP administration
	Reissue 1450 ppm fluoride toothpaste to Arm1 and Arm 3 and 2800 ppm to Arm 2
	Placement of fluoride varnish by trained hygienist to Arm 3
	Complete qualitative interviews with organisational teams
	Brushing charts to be issued
	Collect health care use data for year preceding and intervention year
	Collect resource costs data from the care home
	Completion of data collection forms for resource cost data from the care home/ dental team
Randomisation	Randomisation of homes within 7 days of last baseline visit
3 months from baseline (± 3 days)	Assess changes to mental capacity
	Review concomitant medications
	Adverse event monitoring
	Administer QoL (OIDP and EQ5D survey tools) and relative need (IoRN2)
	IMP administration
	Reissue 1450 ppm fluoride toothpaste to Arm1 and Arm 3
	Reissue 2800 ppm fluoride toothpaste to Arm 2
	Placement of fluoride varnish by trained hygienist to Arm 3
	Brushing charts to be issued
6 months from baseline (± 3 days)	Assess changes to mental capacity
	Examination using ICDAS root caries scoring system undertaken by blinded assessor
	Undertake in depth interviews with residents to consider acceptability, QoL (OIDP and EQ5D survey tools) and relative need (IoRN2)
	Compliance check
	Review concomitant medications
	Adverse event monitoring
	IMP administration
	Reissue 1450 ppm fluoride toothpaste to Arm1 and Arm 3
	Reissue 2800 ppm fluoride toothpaste to Arm 2
	Placement of fluoride varnish by trained hygienist to Arm 3
	Brushing charts to be issued
	Collect health care use data for year preceding and intervention year
	Collect resource costs data from the care home
	Completion of data collection forms for resource cost data from the care home/ dental team
9 months from baseline (± 3 days)	Assess changes to mental capacity
	Review concomitant medications
	Adverse event monitoring
	Compliance check
	Administer QoL (OIDP and EQ5D survey tools) and relative need (IoRN2)
	IMP administration:
	Reissue fluoride toothpastes to control and one intervention arm
	Placement of fluoride varnish by trained hygienist to one arm
	Brushing charts to be issued
12 months from baseline (± 3 days) (end of trial)	Assess changes to mental capacity
	Examination using ICDAS root caries scoring system undertaken by blinded assessor
	Compliance check
	Review concomitant medications
	Adverse event monitoring
	Undertake in depth interviews with residents to consider acceptability, QoL (OIDP and EQ5D survey tools) and relative need (IoRN2)
	Complete qualitative interviews with organisational teams
	Collect health care use data for year preceding and intervention year
	Collect resource costs data from the care home
	Completion of data collection forms for resource cost data from the care home/ dental team

### Statistical analysis

#### Sample size

This is a feasibility study where the primary objective is to assess and estimate the magnitude of feasibility parameters. Consequently, no formal sample size is required for the feasibility parameters. However, assuming a change from baseline to 12 months in the active to arrested mean number of lesions of 0.28 for the control group and a pooled standard deviation of 0.65 [19] and further assuming the experimental interventions are likely to yield at least 50% of the effect of that observed in [19] for the intervention group (i.e. about 0.68 lesions per tooth), based on a reduced concentration (2800  ppm) being used in this study (5000 ppm), the sample size required is 60 per group (180 in total) to achieve at least 84% power to detect a difference (in the change from baseline) of at least 0.4 lesions. For a cluster randomized trial this assumes 3 clusters (2 care homes per group), with an average of 30 subjects per care home, an intra cluster correlation coefficient (ICC) of at least 0.01 and a 2-sided type I error of 5%. Withdrawn patients will not be replaced.

### Qualitative

The process of qualitative data analysis will be iterative process, with transcripts being re-read and reviewed throughout for familiarisation and emergence of new themes to inform and evolve the framework.

The data were summarised and catergorised by themes across the transcripts, whilst being mindful to summarise in way that the context and essence of the participants view is not lost by keeping useful expressions and phrases as much as possible and limiting interpretation.

Finally, a description and explanation of these themes will be developed, by cross examining and interrogating the data, to build emerging theories beyond just the facts. We will focus on the explicit accounts provided by the participants as well the social care perspectives on policy and planning.

### Quantitative

#### Primary outcomes: feasibility

- Number of participants recruited, retained, consented and lost (drop out) through the trial period in each trial arm. This outcome will be summarized by treatment group using descriptive statistics
- Compliance to trial and data collection process by number of examinations completed, mean length of examination, level of supportive need through the trial period in each trial arm. Compliance through a daily diary of the amount of intervention taken will be recorded in a daily diary and summarized over time. The mean compliance will be presented for each treatment group. Where appropriate a model based analysis with average subject compliance as an outcome will be modelled against treatment and stratification variables and considering clustering. A similar approach for the length of examination will also be undertaken. The level of support will be analysed using appropriate categorical methods.
- Fidelity to the trial processes: The number of time teeth brushed with fluoride toothpaste will be presented by treatment group and compared between groups using model based methods. The number of applications of fluoride varnish per participant will also be presented descriptively. Where appropriate, additional model based analyses maybe conducted to determine whether differences are explained by any of the stratification variables

#### Tertiary outcomes: efficacy and economics

- Number and proportion of teeth with decay progression/no change/arrest at tooth surface level between trial arms: The number of teeth with decay progression no change/arrest will be modelled in terms of treatment group and covariates (stratification variables), considering clustering. The mean number of teeth will be presented along with 95% confidence intervals for the difference. The number of teeth with decay progression will be expressed as a percentage and subject to a similar model based approach.
- Statistical Analysis of HRQoL Endpoints: The EQ-5DL will be presented by each of the 5 domains descriptively. In addition, the raw responses will be converted into utilities and the mean difference between treatments presented along with 95% CIs. In addition, the quality adjusted life year (QALY) will be constructed to provide preliminary estimates of QALYs which will also be compared between groups.
- Statistical Analysis of Economic Endpoints: Health resource use will be summarised by treatment group. The costs associated with health resource use will also be summarised.

### Trial data governance

#### Trial management group

A trial management group will convene 3 monthly to monitor trial progress and review any adverse events.

The group will consist of the lead researcher, representative of the CTU, Consultants in Dental Public Health. As deemed appropriate, by the research team a representative from the care homes will also be invited to attend.

### Access to source data and documents

The Investigator(s) will permit trial-related monitoring, audits, REC review, and regulatory inspections by providing the Sponsor(s), Regulators and REC direct access to source data and other documents (e.g. patients’ case sheets, blood test reports, X-ray reports, histology reports etc.).

### Ethics & regulatory approvals

The trial will be conducted in compliance with the principles of the Declaration of Helsinki (1996), the principles of GCP and in accordance with all applicable regulatory requirements including but not limited to the Research Governance Framework and the Medicines for Human Use (Clinical Trial) Regulations 2004, as amended in 2006 and any subsequent amendments.

This protocol and related documents will be submitted for review to The Social Care Research Ethics Committee (SREC), and to the Medicines and Healthcare products Regulatory Agency (MHRA) for Clinical Trial Authorisation

The Chief Investigator will submit a final report at conclusion of the trial to the KHP-CTO (on behalf of the Sponsor), the REC and the MHRA within the timelines defined in the Regulations

### Quality assurance

Monitoring of this trial will be to ensure compliance with Good Clinical Practice and scientific integrity will be managed and oversight retained, by the KHP-CTO Quality Team.

In considering risk relating to the investigational medicinal product (Duraphat® 2800 ppm and varnish) risks to participants would be no higher than that of standard medical care (classified a type A based on MRC/DH/MHRA Joint Project 2011). The licensed medicinal products being used will be used for their licensed range of indications, dosage and form in this study, and so this has been determined as a low-risk trial.

### Data handling

The Chief Investigator will act as custodian for the trial data. The following guidelines will be strictly adhered to:

- Patient data will be pseudo- anonymised*.*
- All pseudo-anonymised data will be stored on a password protected computer.
- All trial data will be stored in line with the Medicines for Human Use (Clinical Trials) Amended Regulations 2006 and the Data Protection Act and archived in line with the Medicines for Human Use (Clinical Trials) Amended Regulations 2006 as defined in the Kings Health Partners Clinical Trials Office Archiving SOP.

### Data management

An electronic CRF will be used provided by King’s College London Clinical Trials Unit. A separate data management plan will be created for the trial.

Confidentiality: The participants will be identified in the database only by initials and a participant study identification number. The study will comply with the Data Protection Act Names and any other identifying detail will NOT be included in any study data electronic file. Anonymised data documents will be stored in a locked cupboard at the sponsor site.

### Insurance/indemnity

King’s College London holds insurance against claims from participants for harm caused by their participation in this clinical study. Participants may be able to claim compensation if they can prove that KCL has been negligent.

### Dissemination

Research in care homes is limited, and in addition very few clinical trials have been conducted in this setting with vulnerable groups. The findings from this study will hold value not only academically, but also to key stakeholder groups including residents, families, care staff, care home and patient representative organisations, dental healthcare providers and commissioners locally and nationally as well as regulators of social care and broader public health landscape.

The working group have developed a dissemination strategy as the trial progresses. The outputs will be based on three broad areas, academic, stakeholder and policy.

The principal outputs for the project will be peer review publications, posters and presentations at regional, national and international conferences, such as European Association for Dental Public Health and International Association for Dental Research.

We will present at meetings and local events with stakeholders, including NHS and Local Authority colleagues to raise the importance of the agenda. We will also send a summary report through a newsletter to all stakeholders involved in the programme, including each care home that has taken part.

It is important that this work is presented at a broader level with a view to inform policy. We will therefore actively seek opportunities to present and publish the work at Public Health England, Chief Dental Officer and NHS meetings and conferences and meetings.

## Discussion

This trial will produce new knowledge and add value to a landscape that is under researched.

Although the efficacy of fluoride interventions is proven, the feasibility of dental research and prevention in this vulnerable group and in the complex care home setting is novel. This work will not only add to our understanding of the interface of dental care and social care but will also contribute to our broader understanding on undertaking research in care home settings. In addition, although the work is set in care homes, there is also applicability of this research to those living in their own homes.

One of the challenges already highlighted in setting up the study is the lack of a coherent structured clear process for setting such trials up. Care home residents not being deemed NHS patients if the research is delivered in their ‘home’ sets up a unnecessary barrier to research in this setting. Equally challenging, is the necessary ethical approval process, that seeks to protect the participant, but can also hinder the value of including all participants in research.

Delivering interventions for vulnerable care home residents, with complex medical and behavioural challenges and high levels of both personal and dental care needs, although clinically beneficial, may not be feasible. The complexities of ethics, consent, capacity and acceptability need to be carefully thought out as to ensure benefit to the residents in their ‘real life’ environment, so that public health programme development is based not only in the science but in the application.

Dental care for older people has been a longstanding issue, and the events of this past year has shone a light on the vulnerabilities of those residing in care homes. In addition, there is a focus on preventative public health and with the fact that dental health and general health are intrinsically linked, this research is landing at a pivotal time.

### Protocol versions

V1.0 Original 26th October 2017.

V2.0 1st January 2019 (change of named statistician).

### Trial sponsor

Sponsor Name: King’s College London.

Address: King’s Health Partners Clinical Trials Office, Floor 16, Tower Wing, Guy’s Hospital, Great Maze Pond, London, SE1 9RT.

Telephone: 020 71885732.

Fax: 020 7188 8330.

Email: helen.critchley@kcl.ac.uk.

### Trial status

The trial was paused due to Covid-19 and data collection points were delayed. We are now discussing re-entering care settings to continue the trial. The study team need to consider recruitment plans and timelines in light of the current status in care homes and the number of losses in the last year due to the pandemic.

## Data Availability

Not applicable.

## Abbreviation

ICDAS: International caries detection and assessment system
